# The factorial survey as an approach to investigate clinical decision-making: examining influences on a clinician's decision to initiate life-sustaining clinical technology for a child with spinal muscular atrophy type 1

**DOI:** 10.3389/fped.2023.1252440

**Published:** 2023-10-24

**Authors:** Mary Brigid Quirke, Lorna Cassidy, Denise Alexander, Cathal Walsh, Katie Hill, Kate Masterson, Nia Flynn, Maria Brenner

**Affiliations:** ^1^ School of Nursing, Midwifery and Health Systems, University College Dublin, Dublin, Ireland; ^2^ Department of Mathematics and Statistics, University of Limerick, Limerick, Ireland; ^3^ Paediatric Intensive Care, Murdoch Children's Research Institute, Melbourne, VIC, Australia; ^4^ School of Nursing, University of Pennsylvania, Philadelphia, PA, United States

**Keywords:** factorial survey, spinal muscular atrophy, long-term ventilation, paediatrics, critical care, children, complex and integrated care needs

## Abstract

**Background:**

Spinal Muscular Atrophy (SMA) type 1 is a debilitating condition with a poor prognosis, though therapeutic advances are promising. Long-term ventilation is a common management strategy as respiratory function deteriorates. Without consensus on best practice respiratory management, the decision to initiate invasive LTV (I-LTV) for this group of young children involves many ethical considerations. Understanding the main influencing factors on a clinician's likelihood to initiative I-LTV for a child with chronic critical illness is important to maintain transparency and trust with the family during this challenging time.

**Methods:**

A factorial survey was used to identify the factors that influence a clinician to support initiation of I-LTV for children with SMA type 1. Factorial survey content was based on literature and evidence-based practice and the content was subject to extensive pretesting and pilot testing. An anonymous survey was disseminated (Oct 2021–Jan 2022), via eight international professional organisations, to clinicians with experience caring for children at the time of initiation of I-LTV.

**Results:**

251 participants answered 514 vignettes on SMA type 1. The greatest influencing factor on clinician's likelihood to initiate I-LTV was parental agreement with the need to initiate I-LTV. Additional qualitative comments from participants support this finding. Clinicians also highlighted the important role of innovative therapies as well as the availability of supports for families when considering initiation however these findings were context based.

**Conclusions:**

The factorial survey approach provides a valuable way of identifying influencers on decision-making in sensitive situations. The findings demonstrate the acceptance of the centrality of parental influence in decisions on care delivery. Effective communication with the child's family is key to ensuring shared understanding and agreement of goals of care. More international research is needed on the long-term effects of novel treatments, as well as impact on quality of life and influence of geographical location, to inform decision-making.

## 1. Introduction

The decision to commence invasive long-term ventilation (I-LTV) for a young child with complex and integrated care needs (CICN) can be a very difficult one for all involved, particularly when the child's diagnosis is likely to mean a poor or uncertain prognosis. Spinal Muscular Atrophy (SMA) type 1, or Werdnig-Hoffmann disease, is an example of such a diagnosis. SMA is a group of hereditary motor neuron diseases estimated to occur somewhere between one in every 3,600–10,000 births (1, 2). SMA type 1 is a serious and most common form of SMA, characterized by muscle weakness, atrophy and hypotonia (especially in the proximal muscles) (3, 4). If not identified through new-born screening, SMA type 1 symptoms are usually evident by six months as infants fail to attain muscular developmental milestones (4). Until recently, the general clinical consensus was that progressive respiratory deterioration will lead to death within two years of age, if technological respiratory supports are not initiated (5, 6). Since 2017, the potential therapeutic landscape for infants diagnosed with SMA has evolved significantly with the ongoing advancement in particular of splicing-modifying therapies (e.g., Nusinersen and gene replacement therapies (e.g., Onasemnogene abeparvovec-xioi) (3). However, while promising, for many families and clinicians caring for an infant with SMA type 1, goals of care discussions continue to be extremely challenging. There is no global agreement on the respiratory management of young children with this diagnosis, and there is not a full global reach of splicing-modifying therapies or gene therapy. The decision to transition from non-invasive ventilation (NIV) to I-LTV in particular can ignite many bioethical challenges (7). The current study focuses on the specific juncture of care, when I-LTV is being considered, and it aims to identify the main influences on clinicians' decisions at this time. Only recently have we begun expanding our understanding about the liminal space between the time initiation is considered and a decision is made (8). In some cases, those involved in the decision-making process (i.e., clinicians, parents, the child) disagree on the pathway of care. Such differences of opinion, if not resolved in a transparent and genuinely collaborative way, can compound the bioethical challenges facing those making the final decision, and thus can have longer-term negative consequences for the individuals involved. Identifying the main influencers on decision-makers in these situations can increase the transparency, and thus trust, between those involved in the decision. We developed a factorial survey to investigate this sensitive topic with clinicians. The factorial survey tool is an innovative form of experimental vignette methodology that is more commonly utilised in behavioural and occupational decision-making research. We make the case that this methodology is also a valuable tool for researching complex and sensitive decision-making in clinical settings. As part of a wider international programme of research into this issue (the TechChild project), the findings from this paper will also provide a basis for the next phase of inquiry, establishing a theory of the initiation of life-sustaining clinical technology for a child in the context of contrasting health, legal, and socio-political systems.

## 2. Materials and methods

### 2.1. Design

**Figure 1:**
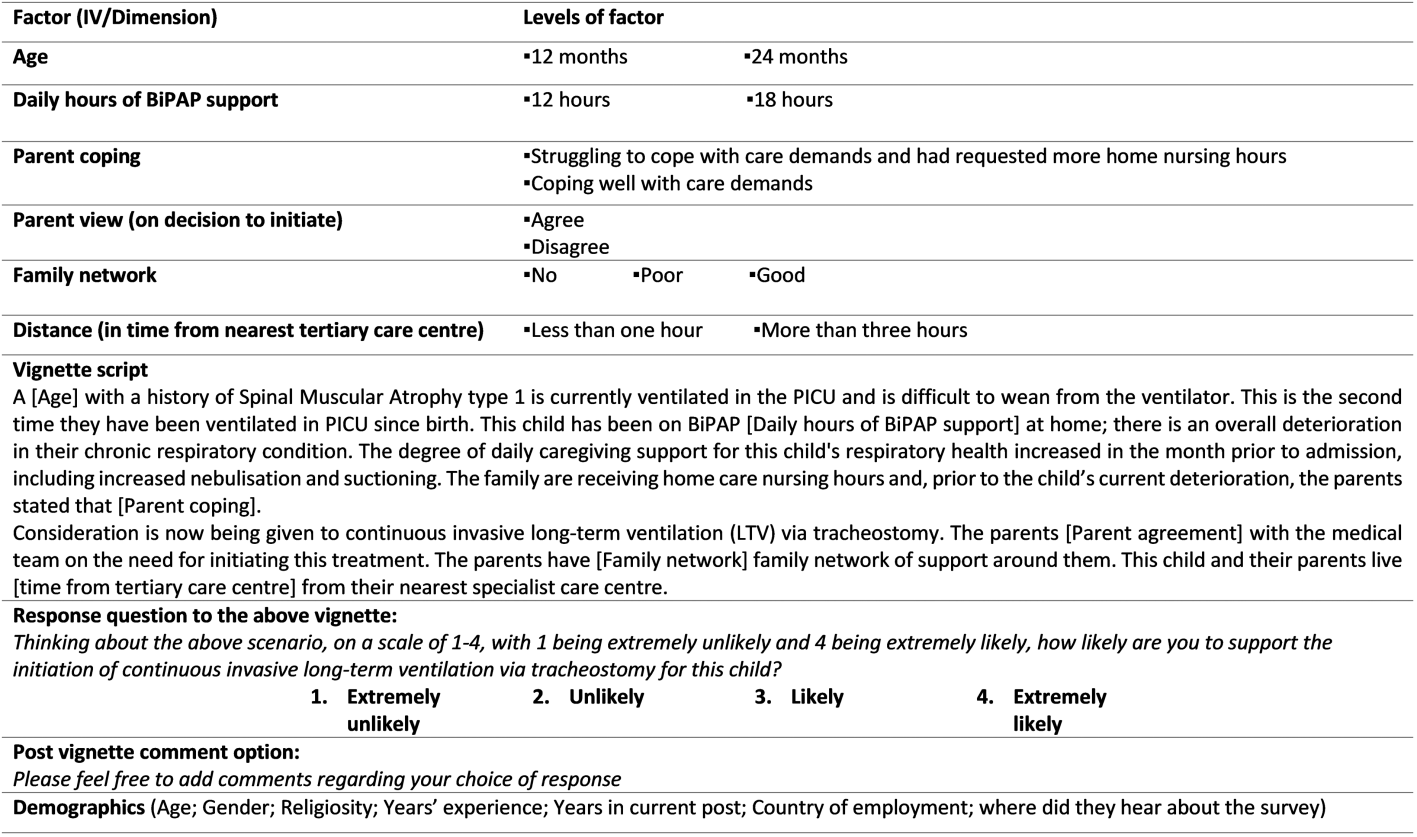
Final SMA type 1 vignette content (factors and levels for infant survey) (extracted from Quirke et al. 2021) (11). Reproduced with permission of the authors. No changes were made. To view the creative commons: http://creativecommons.org/licenses/by/4.0/

A form of hypothetical vignette methodology, the factorial survey, was used to identify the main influences on the decision to initiate I-LTV for a child with SMA type 1. The complexity of measuring the factors associated with the initiation of I-LTV was a key reason to use a factorial survey design. This approach permits interchanging randomisation of each independent variable (IV) level both within and across vignettes as each are presented to a participant (9). This provides greater control to the researcher than a normal survey and thus provides more predictive power for the analysis (10). Therefore, the main influencers on the decision to support initiation can be examined with greater confidence. The development of the cross-sectional TechChild factorial survey followed established guidelines for survey development as well as the factorial survey literature (11). To maximise validity the base content for the survey was extracted from: (1) existing literature on technology dependence and LTV (7, 8, 12); and (2) *n* = 78 interviews with clinicians based on their first-hand experience of similar scenarios. These factors were initially refined in consultation with clinical experts and then further finalised during the development stages of the survey (i.e., cognitive interviewing with members of the target population, statistician consultations, face validity assessment, software randomisation reliability, field and pilot testing). This phase of development was both time and resource intensive (January 2021–September 2021). An in-depth account of the development of the tool is published elsewhere (11). The final online factorial survey consisted of a survey set up on Qualtrics that presented each participant with eight vignettes comprising double-blinded interchangeable factors. Each vignette was followed by a response question offering a forced pseudo-Likert 4-point scale (extremely unlikely, unlikely, likely, extremely likely). The final set of factors and levels, as well as vignette content and response question and scale, are presented in Figure 1 below. The eight vignettes included scenarios with four different exemplar diagnoses (SMA type 1, Bronchopulmonary Dysplasia, Rett Syndrome and Duchenne Muscular Dystrophy). Each participant who completed the survey was presented with approximately two vignettes on SMA type 1 (with allowance for randomisation variability). As set out in Figure 1 the total possible number of scenario combinations (vignette universe) for SMA vignettes was 96 (2 × 2 × 2 × 2 × 3 × 2) (11). At the end of the survey, participants were also asked for basic demographic information to provide context to the responses. After each vignette, respondents were invited to add any comments regarding the vignette or their response. Reporting of the survey in this paper was guided by the Consensus-Based Checklist for Reporting of Survey Studies (CROSS) (13).

The open-ended response section, while optional, was considered an important component to achieve the aim of the study. The topic under investigation is complex and ethically challenging and these comments provided an opportunity for participants to contextualise their responses, or comment on additional influences that they consider important in their decision for each vignette. A qualitative descriptive approach was undertaken with the purpose of providing a descriptive interpretation of participants' views on other important influences relating specifically to the observed vignettes (14, 15). Previous phases of the TechChild study provided an in-depth interpretive exploration of these issues at a broader level (7). The current qualitative component of the study did not seek to replicate this work but instead simply provide additional context to participants' survey responses. This presentation of component of the study was guided by the Critical Appraisal Skills Programme (CASP) checklist (16).

### 2.3. Participants

The target population was defined as multidisciplinary clinicians who have clinical experience of caring for children at the time when initiation of I-LTV is being considered. The sample frame for the survey was the reach of eight large international professional medical organizations (the majority of which are primarily based in USA, Europe, Australia) who agreed to distribute the anonymous survey to their members.

After a process of engagement and organisational board review, the survey and participant documents were approved by each organisational board and distribution methods were guided by the capacity of each organisation involved. In order to achieve the widest reach, non-probability purposive sampling was used; all members of each organisation were eligible to complete the study. The research setting was virtual and Qualtrics survey software (Qualtrics, Provo, UT) was used (17). The survey was distributed anonymously via email, social media (twitter) and/or website/e-newsletter platforms.

Eight vignettes (four vignettes from two age-specific surveys with two differing diagnosis each) were presented via an anonymous online survey to participants. Overall, 277 participants completed 2,056 vignettes for the TechChild factorial survey. Of these, 251 participants answered 514 vignettes for which SMA was the presenting diagnosis (i.e., the focus of the current paper).

The online survey was distributed by the organisations to members between October 2021 and December 2021. Distribution dates varied based on what worked for each organisation. A reminder was sent by the organisation approximately one month after the initial distribution contact. The survey remained open until mid-January 2022. The survey was anonymised at source. The Qualtrics survey protection feature was used to prevent search engine indexing and bot completion. In addition, the study incorporated the recommendations of the RESTORE study to valid participant entries (18). Evidence of one incidence of multiple participation of participants was found and the duplicate submission deleted.

### 2.4. Ethics

Ethical approval was obtained from the research ethics committee at the host institution. All participants were required to read the information sheet and consent form before they could progress to the study. As the study was anonymous, participants were informed that by clicking to progress they were agreeing with the items set out in the consent form. A Data Protection Impact Assessment form for the survey was also approved and deemed low risk by the Data Protection Officer at the host institution. Data was anonymised at source and stored on the Qualtrics server until the survey closed. A data processing agreement was in place with Qualtrics and the host institution. During the data collection period, only the member of the research team involved in data collection had access to the password protected Qualtrics account. When data collection was complete, the data was downloaded to a secured research folder accessible by the research team via encrypted institutional approved computers only and deleted from the Qualtrics platform.

### 2.5. Analysis

Descriptive analysis was used to provide an overview of the participants' profiles. The factors of the vignette as well as demographic factors were the independent variables (IVs). The participant's rating of likelihood to support the decision to initiate was the dependent variable (DV). Each participant contributed up to eight vignettes, however, as each vignette was randomized it could not be considered repeated measures as in a standard survey. Nevertheless, it was likely that a multilevel structure to the data existed where responses were likely to cluster/group within individual respondents. Thus, a mixed effects regression model was deemed the most appropriate approach with vignettes (level 1) and respondents (level 2). Responses to the survey were collapsed into a binary variable (i.e., extremely unlikely/unlikely and likely/extremely likely). A mixed effects binary logistic regression model was then used to identify the main determinants that influence the decision to support I-LTV initiation. Using binary logistic modelling over ordinal logistic modelling provided for a more robust examination, as much less information is required to undertake this type of regression. Categorical predictor variables were dummy coded and statistical modelling was performed using IBM SPSS Statistics for Windows, version 28 (IBM Corp., Armonk, N.Y., USA) (19).

In total, *N* = 277 initiated the survey, *n* = 251 answered at least one vignette question and *n* = 245 completed the entire survey implying a vignette completion rate of 88.5%. Overall, participants completed 1,038 infant vignettes of which 514 pertained to children with SMA type 1. Vignette analysis was completed using listwise deletion of cases when data were missing. In terms of analysis, missing data related to demographic data only. Examination of this data did not demonstrate any particular pattern and was likely the result of dropout prior to completion. Figures concerning each demographic variable are set out in Table 1. Sensitivity analysis was conducted to determine how different values of the IVs impact on the DV. Considering the nested structure of the data, Akaike's information criterion (AIC) was considered most appropriate method to compare predictor models.

**Table 1:** Participant profile.

Profile variable	
Age (*n* = 215)^b^	Mean 45.49 years (SD = 10.32) (Median 44 years; 25–80 years)
Years’ experience with CCCN (*n* = 224)^b^	Mean = 15.8 years; SD = 9.21) (Median = 15 years; 1–50 years)
Gender (*n* = 229)	*n* (%)
Female	138 (60.3%)
Male	87 (38.0%)
Non-binary	1 (.4%)
Prefer not to specify	3 (1.3%)
Belonging to a religious denomination (*n* = 229)	
Yes	119 (52.0%)
No	95 (41.5%)
Not sure	9 (3.6%)
Prefer not to specify	6 (2.4%)
Profession (*n* = 226)	
Medical Doctor	173 (76.5%)
Registered Nurse/Nurse practitioner	33 (14.6%)
Respiratory Therapist	10 (4.4%)
Other members of the MDT^a^	10 (4.4%)
Current country of employment (*n* = 224)^c^	[*Based on UN categorisation system*]
North America	139 (62.1%)
Europe	56 (25.0%)
Oceania	13 (5.8%)
South America	7 (3.1%)
Asia	5 (2.2%)
Africa	4 (1.8%)

^a^ Includes Dietician, Pharmacist, Physician Associate, Physiotherapist/Physical Therapists, Senior Care Assistant.

^b^ Age and years’ experience included two outliers that were removed for vignette related analysis.

^c^ Responses collapsed from countries to continents (as per UN categorisation) to protect participants anonymity.

The qualitative written open-ended response data was examined guided by a qualitative descriptive framework, a well-established approach often used to gain further descriptive insights into a phenomenon (14, 15). An inductive thematic analysis strategy was used informed by the Braun and Clarke framework (20). Considering the nature of the data, this technique was deemed most appropriate for the purpose of analysing patterns, describing the data and interpreting additional contextual information that may have influenced participants responses (20). Assessment of data saturation was not considered relevant in this context. In total, the qualitative data set comprised *n* = 131 vignettes for which participants provided comments (out of a total of 514 SMA vignettes).

## 3. Results

The final sample comprised 251 participants who completed 514 SMA vignettes. Based on those who provided their demographic information, over three quarters of clinicians who participated were medical doctors (76.5%), female (60.3%) with many years of experience of caring for this group of children (Mean = 15.8 years). The majority of the participants worked in the USA (62.1%) and Europe (25.0%), which provides an indication of the reach of the organizations involved in the survey distribution. Table 1 provides an overview of those who took part.

### 3.1. Vignette analysis

Null model (Model 1): In building a multilevel model first we built the unconditional intercept only model, meaning no predictors/IVs were included. Our research question for this model focuses on whether likelihood to support initiation based on a vignette varies across participants (i.e., Does likelihood to support initiation vary across clinicians?). The significant result from the likelihood ratio test between the fixed and random intercept models r (*z *= 3.655, *P *< .001) suggests that the intercept variance varies significantly between respondent units. That is, the observations are nested in participants, providing justification for a multilevel model (21). The interclass correlation coefficient (ICC) for the Null model, which identifies the variation between clusters, indicated that 24.2% of the variability in likelihood to support initiation lies between clinicians.

Predictor model (Model 2): When the predictor factors are included in the model, one of the main effect predictors in the model was observed as significant. The odds of a clinician supporting initiation for a child with SMA type 1 was 8.2 times more likely if parents agree with the decision to initiate compared with if parents did not agree with treatment. The importance of the parental view on the decision is likely to have impacted on the contribution of the other factors i.e., the other factors included, that were considered important in the literature as well as in interviews with clinicians, were not significant. These findings emphasise the central role of parents' involvement in, and support for, the decision to proceed with initiation in this type of scenario. The findings also indicate that, while the other factors are identified as important, parental agreement with treatment is the dominant factor that will likely act as a key influencer on a clinician's decision to support I-LTV for a child with SMA type 1. The other non-significant factors were still included in Table 2 as each factor was considered important based on previous research as well as the experiences of clinicians.

**Table 2:** Overview of the model including contribution of the predictors [guided by LEVEL reporting as recommended by Monsalves et al. (22)].

		Model 1: Null model (*n*_1_ = 514 vignettes *N*_2 _= 251 respondents)	Model 2 *n*_1 _= vignettes 400; *n*_2 _= 194 respondents num^a^)	*P*
		OR (95% CL)	OR (95% CL)	
Vignette factors (level 1)	Value of category	N/A		
Child's age	12 months	N/A	0.268	0.789
	24 months			
BiPAP support	12 h	N/A	0.926	0.355
	18 h			
Parent coping	Coping well with care demands	N/A	0.087	0.931
	Struggling to cope with care demands and had requested more home nursing hours			
Parent view (on decision to initiate)	Agree	N/A	**8.236 (2.96–10.908)**	**<0** **.** **001**
	Disagree			
Family network	A good (network)	N/A	1.752 (1.159–1.724)	0.082
	No/A poor (network)			
Distance (from nearest tertiary care centre)	Less than one hour	N/A	0.108 (0.588–1.031)	0.914
	More than three hours			
Respondent factors				
Age (of clinician)		N/A	0.359 (−0.058–0.084	0.720
Gender	Male	N/A	0.158 (−0.660−0.775)	0.875
	Female			
Religiosity	Yes	N/A	−0.658 (−0.918–0.458)	0.511
	No			
No of years working with children with complex medical needs		N/A	0.056 (−0.077–0.081)	0.955
Interclass Correlation Coefficient (ICC)		0.242 (<0.001)	NA	
Akaike Information Criterion (AIC)		2227.17	1864.737^b^	

Bold value indicates significant at *P* = 0.001.

^a^ Note that the number of respondents in this model is lower due to non-response to one of the demographic questions included.

^b^ An intermediate model was build using only the vignette predictor variables however, whilst the sample was larger (i.e., missing data was primarily from demographic data, little difference were observed between this intermediate model so this data were not included. AIC also indicated better fit with demographics included than with just vignette factors (2345.51).

### 3.2. Analysis of comments

**Figure 2:**
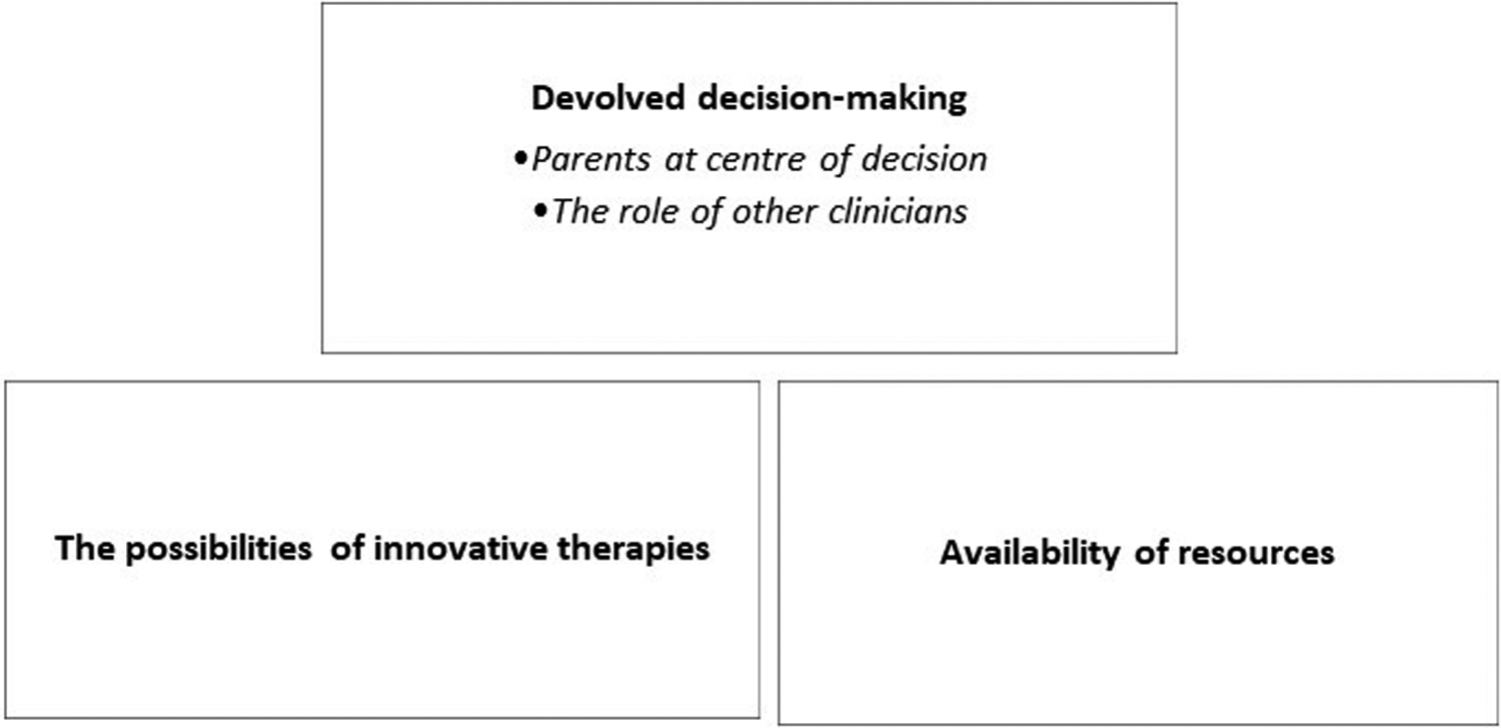
Themes from participants responses to the open-ended comment section of the survey.

The aim of the qualitative analysis was to describe the additional information offered by participants. Three semantic themes emerged from this data (Figure 2).

#### 3.2.1. Theme one: devolved decision-making

##### 3.2.1.1. Parents at centre of decision

Many clinicians stated that parents and families were central to decision-making, a finding that is reflected in the analysis of the factorial survey. In scenarios that indicated a parental lack of support for initiation, some clinician's indicated that further discussions around goals of care and ceilings of care were needed. A focus on gaining consensus with families was also noted by some participants. However, whether the clinician's view on the decision to initiate I-LTV was congruent with the family's position or not, it was evident that a priority for many clinicians was that families understood what the decision meant either way. Communication with the family was also emphasized as essential to effective care. In terms of the clinician's choice of response to the vignette item, consideration of this issue seemed (1) confirmatory for those clinicians in alignment with parents' decision or (2) referenced as a reason for accepting the parents’ choice over their own preference for treatment.

*“I would counsel the family against it, but ultimately, if I felt like they understood, wouldn’t stop it. But would never want [this initiation of I-LTV] for my child [in this scenario]”. [Physician, USA]*

*“The [initiation] of I-LTV for a life limiting disease where life may be prolonged by an immensely expensive drug but where the child will still need 24-hour care means that parents must agree with I-LTV. These parents do not” [Physician, Europe]*

*“If the parents disagree with the choice of a tracheostomy, I would ensure they understand the consequences and likely further deterioration of the child. I would prepare them for the eventuality that their child could die at home or in the hospital”. [Nurse, USA]*

##### 3.2.1.2. The role of other clinicians

Some participants provided a response to the vignette and then went on to state that while this was their opinion, they did not feel that the decision could be made in the absence of wider multidisciplinary consideration. Obtaining perspectives of the wider multidisciplinary team or consulting with specialist consultants (e.g., neurology or respiratory) were indicated as factors that would contribute to their decision.

*“I would defer to a paediatric Neurologist but my inclination would be not to consider I-LTV. It is however an MDT decision and I could be persuaded otherwise” [Physician, Europe]*

What is more, for those assured in their decision, their comments sometimes suggested that, when considering the decision to initiate, clinicians were already mindful of the need for, and availability of, other important services such as palliative care. While not explored in this study, this highlights that the presence orlack of such services and supports may also contribute to a clinician's likelihood to support initiation or not.

*“Ethics and palliative team would likely need to be involved and detailed family meeting would need to be done. If parents are aware of outcomes of not choosing tracheostomy in this situation, [services] such as hospice, death, palliative support can be provided to the family”. [Physician, USA]*

#### 3.2.2. Theme two: the possibilities of innovative therapies

Consideration of the use of novel therapies were noted by a number of participants, primarily with reference to Nusinersen. Clearly, such therapies are a major factor for some clinicians when considering care and treatment pathways for these children.

*“In the absence of this type of treatment, this child will be condemned to be a body on a ventilator unable to move. I do not think this is a good quality of life for the child or the family and this is how I would counsel the family”. [Physician, USA]*

While the advent of new therapies was mentioned, the majority of participants suggested that the reach of new therapies remained limited globally in terms of access. While not explicitly measured, the international reach of this study and comments which noted their own regional context, highlights how geographical location may influence treatment options for children with SMA type 1.

*“This is a difficult question to answer, because a similar child in England would be on Nusinersen or be offered gene therapy. If on Nusinersen and failing from a respiratory point of view leading to a trachy, that would mean they would no longer meet the criteria for Nusinersen and so would lead to a conversation with the parents about [different options]” [Physician, USA]*

*“After Nursinersen era we have a few patients in a similar condition. All of them still on NIV even without home nursing hours. In our area, previously these families usually refused tracheostomy due to the poor expected quality of life. Those patient non-responders to the current therapy are not encouraged to move to tracheostomy”. [Physician, Europe]*

Some respondents also indicated that they would be curious as to the potential for such treatments but, accessible or not, acknowledged that restrictions on age, distance from a treatment centre, as well as tolerance and appropriateness for the individual child would still need to be considered, thereby highlighting the limitations of such treatment for many patients.

*“Nusinersen may be a game changer for these children although my understanding is that it is only available to children less than 12 months of age” [Physician, Europe]*

#### 3.2.3. Theme three: availability of resources for family support

In addition to the availability of specific innovative therapies, participants noted the importance of other resources with particular emphasis on home care packages and other supports for the family, especially those without a family network. Indeed, participants from some areas noted regional policies preventing them from even offering I-LTV to these patients with SMA type 1 highlighting again differences in care depending on location.

*“I would support this intervention if resources allow, although in my Center we would not offer this course of treatments, given the likely poor outcome and poor quality of life of a child with SMA1” [Physician, Africa]*

*“Much may depend on the support services that the family receives. Who pays for services?” [Physician, USA]*

*“In the country I am in we would support these children with non-invasive but not invasive home ventilation unless briefly for intercurrent illness” [Physician, Oceania*]

## 4. Discussion

The findings of this factorial survey clearly highlight the central role parents play in decision-making at this difficult juncture in a child's care pathway. The consequences of the decision will, in most instances, mainly be felt by the family; a consideration further reflected by the clinicians in their comments. The burden of care on families of children initiated on I-LTV is well recognised (23–27), and when a family and clinician have different views on this decision, ethical considerations are brought to the forefront. Incongruence between the parent and clinician view was rarely observed in the current context. Where it was observed, clinicians generally contextualised their response with comments noting the importance of further discussions for all involved. In the main, the focus of discussions were framed in terms of clarifying that the family's decision were aligned with their goals of care and/or ensuring families understood the consequence of their decision, while for a few there was more emphasis on gaining consensus. The qualitative findings in this study highlight the importance of effective communication and clinicians’ time spent with the family with the objective of establishing a shared understanding and resolution around goals of care. Overall, these findings also reflect an awareness of a shift from paternalistic decision-making approach to a shared or family orientated approach observed in the literature (28). In terms of improving quality of care, this study underlines the importance of establishing and/or enhancing resources to support clinicians to better communicate with families, build genuine rapport and maintain trusting relationships within the context of local organisational and legal parameters (24).

While the parental view was the only significant factor to predict a clinician's likelihood to initiate I-LTV based on the vignettes presented; the non-significant influence of other factors measured were nevertheless interesting. For example, while nearly a quarter of the variance lay between clinicians' demographic factors (such as clinicians' gender, age, years' experience, religious affiliation or not), this did not significantly contribute to the model. In the literature some evidence indicates that personal and professional factors may play a role in such decision-making, but the data is less clear (8, 29). In addition to demographic factors, previous inquiry and scientific literature demonstrates the importance of the other vignette factors, however this study showed that when a clinician reads the parents perspective on initiation of I-LTV for children with SMA type 1, this seems to be the main driving influence.

Following extensive consultation with expert clinicians when developing the survey, innovative therapies were not included in the vignettes. This was primarily because of the age profile of the case studies, as well as variability in terms of their use and availability (or lack of) both within and between countries. Moreover, the diagnosis of Spinal Muscular Atrophy (SMA) type 1 was used as one of four exemplar diagnoses for the purpose of investigating this sensitive decision-making topic with clinicians. The qualitative data reflected how new treatments are changing perspectives on treatment options for these children, especially in the US. The advancement of splicing-modifying therapies and snm1 gene replacement therapies in particular have demonstrated positive outcomes as well as major potential especially for children with SMA 1 and 2 (3, 30). However, while promising, the long-term benefits of treatment have yet to be established and no global consensus on recommended treatment plans currently exists. Clinical limitations, side effects and contra-indications are still under investigation and it is clear that to date such treatments do not offer a panacea for SMA type 1 (3, 6, 26, 31, 32). In addition, for many countries outside the USA and UK these innovative treatments remain inaccessible or restricted due to financial constraints (26), a fact also noted by some participants in their comments. Clinicians' understanding of SMA prognosis may be evolving, and, assuming these therapies continue to shift the landscape of SMA prognosis, it would be interesting to assess the impact of the broader use of novel therapies on the leading influence of the parents view on a clinician's decision to initiate. Similarly, the access and availability of other resources and services particularly palliative care and home care packages are important considerations for many clinicians when deciding to support initiation or not, especially in terms of geographic location. Only a small number of clinicians from developing countries participated in the study and where these clinicians commented, the limited facilities of the care centre as well as the resource capacity of the family was noted as an influencing factor in treatment for children with SMA type 1.

Indeed, previous empirical reviews on this topic have noted the dominance of USA literature in the area of decision-making around technology dependence (28). In the current study, substantial efforts were made to include a global response through the distribution of the survey via eight international professional organisations whose membership comprises a broad range of specialisms and disciplines. Nevertheless, the majority of respondents reported working in settings based in either USA or Europe. Comments from the small cohort of respondents from more diverse geographical settings suggest that more detailed examination of broader cohorts would contribute to a wider perspective on how treatment availability and regional resource issues influence clinical decisions of care for children with SMA type 1.

As far as the authors are aware, this is the first study to utilise a factorial study design in a critical care setting. While it is never possible to include in a survey every detail that may contribute to a clinician's decision to initiate I-LTV, the external validity of the study was greatly strengthened through the use of evidence-based vignette content, alongside a detailed approach to survey development (11). The inclusion of the qualitative component provided further opportunity for the clinicians themselves to identify any additional factors that may influence decision-making. The main influencing factors identified in this paper form a clear picture of the driving influences on a clinician's decision to initiated life sustaining technology dependences for a child with SMA type 1. Identifying influencers on decision-making in these situations can improves transparency in the decision-making process and can contribute the fostering of positive relationships between those involved. Using a diagnosis of Spinal Muscular Atrophy (SMA) type 1 as an exemplar diagnosis, this factorial survey approach had the capacity to examine this challenging topic in a more sensitive way with clinicians. Following on from this work, analysis of the different case diagnoses and clinical disciplines and further exploration of the main drivers identified will be undertaken to build consensus as well as develop our understanding of the broader social and legal influences in play.

## 5. Conclusions

The factorial survey is a valuable approach to researching complex and sensitive decision-making in clinical settings. In our examination of the initiation of I-LTV for a child with SMA type-1, it is evident that the decision to initiate (or not) can be complex and involve many bioethical considerations, especially in the light of evolving advances in terms of potential treatment options and a lack of consensus regarding best management practice. However, with the lack of accessibility of novel treatments in many settings and restrictions regarding the appropriateness of such treatments for many children, the main influence on a clinicians' decision whether or not to initiate centres around the family perspective and consensus of support for such treatment. Facilitating opportunities for effective communication as well as the development of trusting relationships and between families and those involved in their care is essential in achieving a family's value-aligned goals of care for their young child with SMA type 1.

## Data Availability

The raw data supporting the conclusions of this article will be made available by the authors, without undue reservation, at the discretion of the corresponding author.
